# Nicotinic Acetylcholine Receptors Are Novel Targets of APETx-like Toxins from the Sea Anemone *Heteractis magnifica*

**DOI:** 10.3390/toxins14100697

**Published:** 2022-10-11

**Authors:** Rimma S. Kalina, Igor E. Kasheverov, Sergey G. Koshelev, Oksana V. Sintsova, Steve Peigneur, Ernesto Lopes Pinheiro-Junior, Roman S. Popov, Victoria E. Chausova, Margarita M. Monastyrnaya, Pavel S. Dmitrenok, Marina P. Isaeva, Jan Tytgat, Sergey A. Kozlov, Emma P. Kozlovskaya, Elena V. Leychenko, Irina N. Gladkikh

**Affiliations:** 1 G.B. Elyakov Pacific Institute of Bioorganic Chemistry, Far Eastern Branch, Russian Academy of Sciences, 690022 Vladivostok, Russia; 2 Shemyakin-Ovchinnikov Institute of Bioorganic Chemistry, Russian Academy of Science, 117997 Moscow, Russia; 3 Toxicology and Pharmacology, Campus Gasthuisberg, University of Leuven (KU Leuven), 3000 Leuven, Belgium

**Keywords:** sea anemones, phylogeny, APETx-like toxins, nicotinic acetylcholine receptors, acid-sensing ion channels

## Abstract

The nicotinic acetylcholine receptors (nAChRs) are prototypical ligand-gated ion channels, provide cholinergic signaling, and are modulated by various venom toxins and drugs in addition to neurotransmitters. Here, four APETx-like toxins, including two new toxins, named Hmg 1b-2 Met_ox_ and Hmg 1b-5, were isolated from the sea anemone *Heteractis magnifica* and characterized as novel nAChR ligands and acid-sensing ion channel (ASIC) modulators. All peptides competed with radiolabeled α-bungarotoxin for binding to *Torpedo californica* muscle-type and human α7 nAChRs. Hmg 1b-2 potentiated acetylcholine-elicited current in human α7 receptors expressed in *Xenopus laevis* oocytes. Moreover, the multigene family coding APETx-like peptides library from *H. magnifica* was described and in silico surface electrostatic potentials of novel peptides were analyzed. To explain the 100% identity of some peptide isoforms between *H. magnifica* and *H. crispa*, 18S rRNA, COI, and ITS analysis were performed. It has been shown that the sea anemones previously identified by morphology as *H. crispa* belong to the species *H. magnifica*.

## 1. Introduction

For many years, the family of nicotinic acetylcholine receptors (nAChRs) has been the focus of researchers’ interest as a vital target for the critical neurotransmitter acetylcholine (ACh), novel drugs (e.g., myorelaxants, analgesics, and neuroprotective agents) and natural toxins from bacteria, algae, plants, and animals [1,2,3]. The identification of nAChRs as the first neurotransmitter receptors has major practical consequences from the perspective of protein receptors targeting for therapeutic intervention and conception of allosteric modulation, including further development of allosteric pharmacological agents [4,5]. nAChRs belong to the Cys-loop superfamily of pentameric ligand-gated ion channels. In mammals 16 nAChR subunits (α1–10, β1–4, γ, δ, and ε) form non-muscle receptors: homo-pentamers of α7, α8, and α9, hetero-pentamers of α2–α6 combined with β2–β4, or α7 with β2, and α9 with α10 subunits, and in muscles hetero-pentameric receptors composed of two α1, one β1 and δ, plus a fetal subunit γ further replaced by adult ε subunit [3,4]. nAChRs provide fast excitatory neurotransmission or neuromuscular transmission in both central and peripheral nervous systems and play pivotal roles in the etiology of neurological disorders. The diversity of nAChRs subtype composition, specific biophysical properties and localization make it difficult to pharmacologically regulate their functions [3,4,5].

Today, a wide diversity of ligands affecting nAChRs has been described. The low-molecular-weight compounds (quaternary ammonium salts, alkaloids, heterocyclic compounds, etc.) [2], synthetic oligoarginine peptides [6,7] and venom peptides of various structural classes: three-finger toxins [3,8] and phospholipases A2 from snakes [2,9], α-conotoxins (α-CTx) [3,10], potassium channel scorpion toxins of α family (α-KTx) [11], toxin-like Ly6 [12] and C-type lectin-like proteins [2], were shown to act as agonists, antagonists, blockers, positive or negative allosteric modulators of distinct nAChR subtypes. The most investigated and numerous peptide modulators of nAChRs are snake three-finger α-neurotoxins and α-CTx [3,10]. The first of them contributed substantially to the nAChRs characterization [8,13] while α-CTx promoted the current theoretical framework for ligand recognition of the muscle and non-muscle nAChRs [3,10,14].

So far, there has been only one report about a high-molecular-weight toxin Condytoxina 2 from the sea anemone *Condylactis gigantea* with unspecified sequence that affected cholinergic responses of snail *Zachrysia guanensis* and mice neurons. This toxin was shown to act as a noncompetitive antagonist at concentrations up to 25 nM and potentiate nicotine-induced current at higher concentrations [15]. Therefore, to date no sea anemone peptides with complete structure determined have been published as ligands to nAChRs.

Here, we report the structure and biological effect of APETx-like peptides (a well-known fold of sea anemone toxins) as inhibitors of ASICs as well as novel nAChRs ligands. Among isolated peptides, two peptides, named Hmg 1b-2 and Hmg 1b-4, were identical to *H. crispa* toxins Hcr 1b-2 and Hcr 1b-4, previously shown to inhibit ASIC1a and inhibit (Hcr 1b-2) or potentiate (Hcr 1b-4) ASIC3 channels [16,17]. ASICs are ligand-gated trimeric cation channels, activated by the proton concentration increase [18,19,20]. In the list of ASIC isoforms, ASIC1a and ASIC3 are the most abundant and functionally important [18,19]. ASICs activation causes a Na^+^ (ASIC1a and ASIC3) and Ca^2+^ (ASIC1a) influx leading to cell membrane depolarization and neurodegeneration, respectively [19]. They are widely expressed in the peripheral nervous system (ASIC1a and ASIC3) and central nervous system (ASIC1a), contribute to nociception and co-localize with a large number of nociceptive receptors. Currently ASICs are considered as a promising target for pain and neurodegeneration management [18,19].

In addition, we have studied APETx-like peptides binding both to muscle-type nAChR of *Torpedo californica* and human α7 nAChR. Moreover, we found out several APETx-like peptide isoforms, in addition to the three non-modified ones, using a PCR technique termed rapid amplification of cDNA ends (RACE). We assembled full transcript sequences for Hmg 1b-2 and Hmg 1b-5, as well as determining a gene encoding sequence for Hmg 1b-5. These results point to the existence of an APETx-like peptide combinatorial library in *H. magnifica*. We also revealed and discussed the reason for the 100% identity of some isoforms between *H. magnifica* and *H. crispa.*

## 2. Results

### 2.1. Activity-Guided Isolation of Toxins from H. magnifica

**Figure 1:**
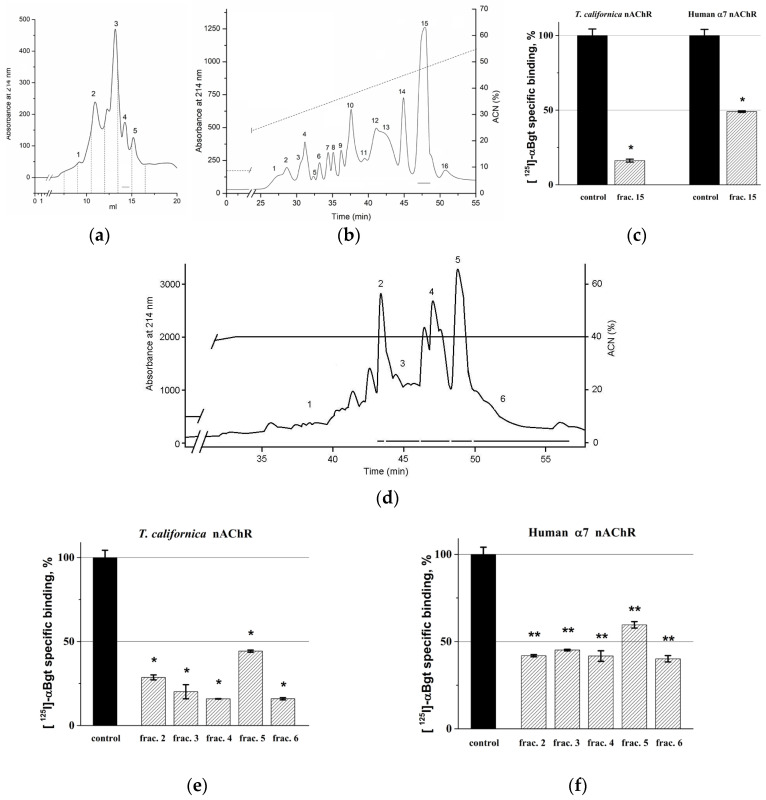
Activity-guided isolation of *H. magnifica* toxins. (**a**) Size exclusion chromatography of the 40% hydrophobic fraction after Polychrome-1 on a Superdex Peptide 10/30 column eluted with 10% acetonitrile (ACN) in 0.1% trifluoroacetic acid (TFA). The numbers indicate collected and tested fractions. Active fractions are accentuated by solid lines. (**b**) RP-HPLC of fraction 4 after size exclusion chromatography on a Luna C18 (10 mm × 250 mm) column in a gradient of acetonitrile in 0.1% TFA. The numbers indicate collected and tested fractions. Active fractions are accentuated by solid lines. (**c**) Competition of fraction 15 from (**b**) (0.1 mg/ml) with [^125^I]-αBgt for binding to muscle-type *T. californica* and human α7 nAChRs (average ± SEM value from three measurements; the black bars correspond to the control 100% of the specific binding of the radioligand to the respective receptor (calculated as the difference between total binding and non-specific binding in the presence of a large excess of α-cobratoxin). *, Fraction 15 showed significant inhibition both on *T. californica* and human α7 nAChRs with *p* = 0.000146 and 0.00102, respectively, in the one-way ANOVA with the Tukey post-hoc test versus respective Control. (**d**) RP-HPLC of fraction 15 on a Luna C18 (10 mm × 250 mm) column in 40% of acetonitrile in 0.1% TFA. The numbers indicate collected and tested fractions. Active fractions are accentuated by solid lines. (**e**,**f**) Competition of fractions 2–6 from (**d**) (0.1 mg/ml) with [^125^I]-αBgt for binding to muscle-type *T. californica* (**e**) and human α7 nAChRs (**f**) (average ± SEM value from three measurements). All fractions showed significant inhibition with *p* < 0.0001 (*) or *p* < 0.0005 (**) in the one-way ANOVA with the Tukey post-hoc test versus respective Control.

The search for active peptide components in the mucus of *H. magnifica* which could compete with radiolabeled α-bungarotoxin ([^125^I]-αBgt) for binding to nAChRs(α1β1γδ muscle-type from *Torpedo californica* ray electric organ membranes and human α7 subtype transfected in GH_4_C_1_ cells) was carried out according to a well-established scheme combining multi-stage fractionation including hydrophobic, size exclusion chromatography, and reverse-phase high pressure liquid chromatography (RP-HPLC) [21] with the measurement of biological activity by radioligand assay. The total active hydrophobic fraction [21] and the subsequent fraction 4 (Figure 1a) inhibited specific binding of [^125^I]-αBgt to the muscle-type *T. californica* nAChR by about 20% (data not shown). As a result of the first RP-HPLC round of fraction 4 (Figure 1b), 16 fractions were obtained and only fraction 15 inhibited specific binding of [^125^I]-αBgt to the muscle-type *T. californica* nAChR and human α7 nAChR (Figure 1c). The second round of RP-HPLC of the fraction 15 produced six fractions (Figure 1d), five of them (2–6) at a concentration of 0.1 mg/mL inhibited the [^125^I]-αBgt-specific binding to both receptors by 55–85% and 39–61%, respectively (Figure 1e,f). Fraction 1 was excluded from the analysis because of the natural product amount limitation.

**Figure 2:**
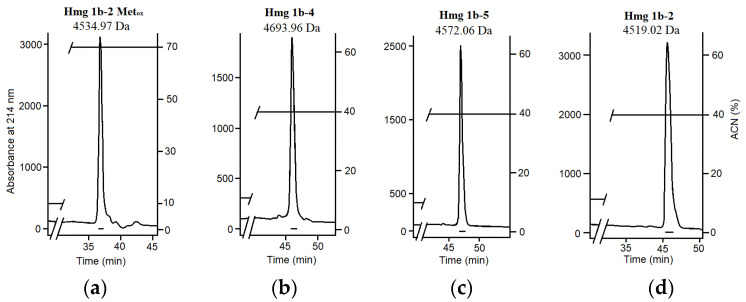

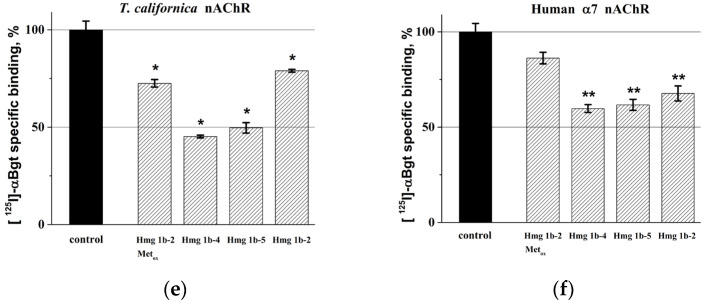
Activity-guided isolation of *H. magnifica* toxins (continued). (**a**–**d**) RP-HPLC of major peptides from fraction 2 (Hmg 1b-2 Met_ox_) (**a**), fraction 4 (Hmg 1b-4 and Hmg 1b-5) (**b**,**c**), and fractions 5 and 6 (Hmg 1b-2) (**d**) on a Luna C18 column (10 mm × 250 mm) in a gradient of acetonitrile in 0.1% TFA. (**e**,**f**) Competition of purified toxins Hmg 1b-2, Hmg 1b-2 Met_ox_, Hmg 1b-4, and Hmg 1b-5 with [^125^I]-αBgt for binding to muscle-type *T. californica* at 20 μM concentration (**e**) and human α7 nAChRs (**f**) at 40 μM concentration (average ± SEM value from three measurements; the black bars correspond to the control 100% of the specific binding of the radioligand to the respective receptor (calculated as the difference between total binding and non-specific binding in the presence of a large excess of α-cobratoxin). *, All toxins (Hmg 1b-2 Met_ox_, Hmg 1b-4, Hmg 1b-5 and Hmg 1b-2) showed significant inhibition with *p* = 0.00323, 0.0001204, 0.0001824 and 0.01076, respectively, in the one-way ANOVA with the Tukey post-hoc test versus Control; **, Hmg 1b-4, Hmg 1b-5 and Hmg 1b-2 showed significant inhibition with *p* = 0.00207, 0.0026 and 0.00567, respectively, in the one-way ANOVA with the Tukey post-hoc test versus Control.

At the final step of the purification, peptides with monoisotopic molecular masses of 4534.97 Da from fraction 2 (named Hmg 1b-2 Met_ox_—see further), 4693.96 and 4572.06 Da from fraction 4 (Hmg 1b-4 and Hmg 1b-5), and 4519.02 Da from fractions 5 and 6 (Hmg 1b-2) were obtained (Figure 2a–d). Fraction 3, containing peptides with molecular masses in the ranges of 4500–4760 and 5400–5870 Da according to MALDI MS analysis, was not taken for further studies.

The monoisotopic molecular masses of peptides, isolated from fractions 4 (4693.96 Da, measured), 5 and 6 (4519.02 Da, measured), perfectly matched with those of APETx-like peptides Hcr 1b-4 (4693.94 Da, theoretical) and Hcr 1b-2 (4519.00 Da, theoretical), previously derived from the sea anemone *H. crispa* [16]. RP-HPLC co-injection of these peptides with respective native Hcr 1b-2 and Hcr 1b-4 in a 1:1 ratio gave a single symmetrical peak each, which allowed us to conclude that the peptides were identical. The molecular masses of other peptides from fractions 2 (4534.97 Da) and 4 (4572.06 Da) did not correspond to any of those described.

All purified toxins from *H. magnifica*, HmgTxs, inhibited [^125^I]-αBgt-specific binding with both *T. californica* muscle-type and human α7 nAChRs (Figure 2e,f). The most active toxins turned to be peptides from fraction 4 (Hmg 1b-4 and Hmg 1b-5), which inhibited radioligand binding to these receptors by 50–55% and 38–40% at 20 and 40 μM, respectively (Figure 2e,f).

### 2.2. Peptide Sequences Determination

To determine the sequence, each alkylated peptide was digested with cyanogen bromide and resulting fragments were separated by RP-HPLC [16]. Peptide sequences were determined using high-resolution tandem mass spectrometry (MS/MS). All detected monoisotopic masses of molecular ions of polyprotonated peptides accurately correlated with theoretical values calculated from proposed sequences. Since CID MS/MS method does not distinguish between isobaric Leu and Ile residues, Ile/Leu assignment was based on homology with Hcr 1b-1–Hcr 1b-4 [16,22].

**Figure 3:**
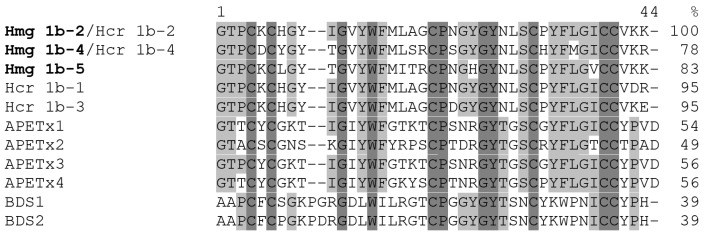
Multiple sequence alignment of the APETx-like peptides: Hcr 1b-1 (P0DL87), Hcr 1b-2 (=Hmg 1b-2) (C0HL52), Hcr 1b-3 (C0HL53), and Hcr 1b-4 (=Hmg 1b-4) (C0HL54) from *H. crispa*; Hmg 1b-5 (C0HLS4) from *H. magnifica*; APETx1 (P61541), APETx2 (P61542), APETx3 (B3EWF9), and APETx4 (C0HL40) from *Anthopleura* *elegantissima*; BDS1 (blood depressing substance 1) (P11494) and BDS2 (P59084) from *Anemonia* *sulcata*. Identical and conserved amino acid residues are shown on a dark and light gray background, respectively. Vector NTI Advance ™ 11.0 (Invitrogen, Carlsbad CA, USA) [23] was used for multiple sequence alignment.

The sequences of peptides with molecular masses of 4693.96 (Figure 2b) and 4519.02 Da (Figure 2d) were shown to be completely identical to Hcr 1b-4 and Hcr 1b-2, respectively (Figure 3), as it was proposed based on their monoisotopic masses and chromatographic co-elution. Consequently, they were named Hmg 1b-4 and Hmg 1b-2, respectively. Sequences of N-terminal (1–16 aa, 2022.91 Da, detected monoisotopic) and C-terminal (17–41 aa, 3102.47 Da) fragments of Hmg 1b-2 were determined based on b_2_–b_14_ ions (N-termini) and b_2_–b_13_, y_1_–y_15_ ions (C-termini) in the CID spectra of doubly charged precursor ions (data not shown). Hmg 1b-4 sequence was elucidated from spectra of polyprotonated alkylated peptide (1–41 aa, 5330.33 Da) where b_2_, b_4_–b_15_, and y_1_–y_8_, y_10_–y_17_ ions were detected, as well as from spectra of peptide C-terminal fragment (17–34 aa, 2259.01 Da) where ions b_3_, b_4_ and b_6_–b_16_ were detected (data not shown).

**Figure 4:**
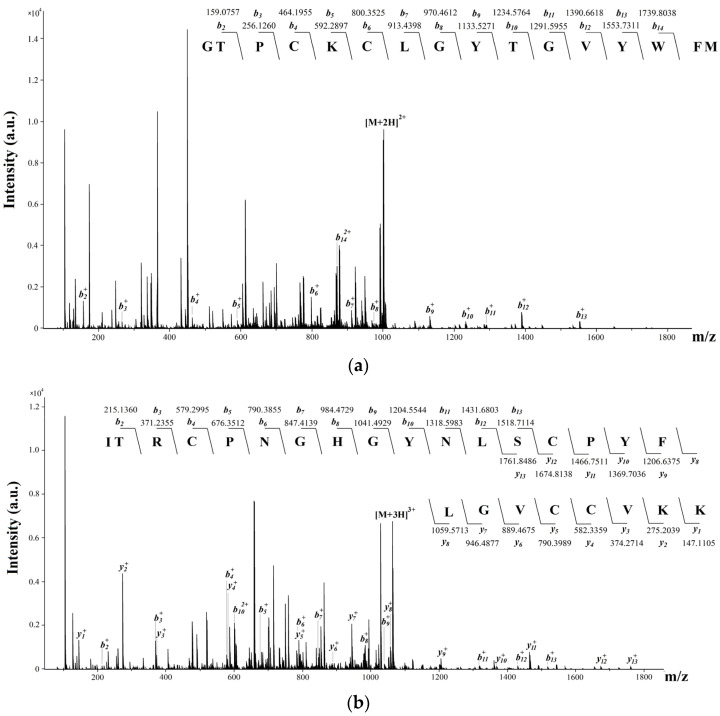
ESI MS/MS spectra derived from polyprotonated N- and C-terminal fragments of alkylated peptide Hmg 1b-5. CID spectra of [M+2H]^2+^ molecular ion (1–16 aa, 2004.91 Da) (**a**) and [M+3H]^3+^ molecular ion (17–41 aa, 3191.52 Da) (**b**). Identified y- and b-type ions series are noted above the mass spectrum. Amino acid residues are indicated by capital letters.

The amino acid sequence of the peptide with molecular mass of 4572.06 Da (Figure 2c) was elucidated from the CID spectra of molecular ions of the polyprotonated alkylated peptide (1–41 aa, 5208.45 Da, b_2_–b_14_ and y_1_–y_13_ identified ions) and two of its fragments (1–16 aa, 2004.91 Da, b_2_–b_14_ identified ions, and 17–41 aa, 3191.53 Da, b_2_–b_13_ and y_1_–y_13_ ions) (Figure 4). The novel peptide was found to be a close homolog of APETx-like peptides Hcr 1b-1–Hcr 1b-4 from *H. crispa* (Figure 3) and it was named Hmg 1b-5.

The sequence determination of the peptide with molecular mass of 4534.97 Da (Figure 2a) was performed similarly. Analyses of CID spectra derived from polyprotonated alkylated peptide (1–41 aa, 5171.36 Da, b_2_–b_15_ and y_1_–y_15_, y_17_ identified ions) showed that N- and C-terminal sequences are identical to those of Hcr 1b-2 (1–15 aa and 25–41 aa). The alkylated peptide was not digested with cyanogen bromide. It supports the idea of oxidized Met in the peptide structure rather than substitution, since theoretical monoisotopic molecular mass of derivative with an oxidized methionine, Hmg 1b-2 Met_ox_ (4534.99 Da) was in perfect agreement with the detected mass of the investigated peptide (4534.97 Da). Therefore, we concluded that the main component of fraction 2 was Hmg 1b-2 Met_ox_.

### 2.3. Electrophysiological Effects of Hmg 1b-2 on nAChRs

**Figure 5:**
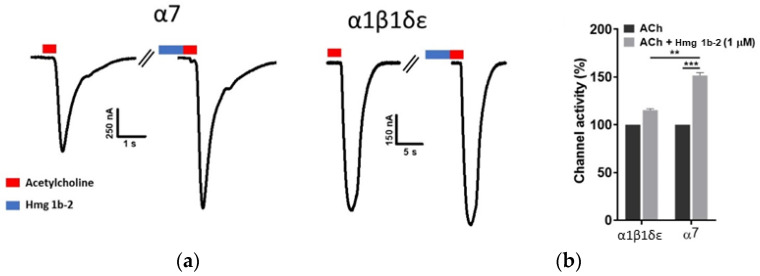
(**a**) Electrophysiological characterization of Hmg 1b-2 (1 µM) effect on human α7 and α1β1δε nAChRs expressed in *X. laevis* oocytes. The receptors were gated by a variable time duration pulse of ACh (red blocks, 100 µM for α7, and 500 µM for α1β1δε). The first and the second peak amplitude represent the absence (control) and presence of 1 µM of Hmg 1b-2 (blue blocks), respectively. The toxin was applied for 60 s, immediately followed by a pulse of ACh. (**b**) Comparative analysis of the channel activity in the absence and presence of Hmg 1b-2 (*n* = 4) ± SEM; SEM: standard error of the mean. ** *p* < 0.01; *** *p* < 0.001.

To explore the effect of the peptide Hmg 1b-2 on nAChRs activity, electrophysiological measurements were performed on human α7 and muscle α1β1δε receptors heterologously expressed in *Xenopus laevis* oocytes. At concentration of 1 µM, Hmg 1b-2 had no activating effect on tested nAChRs when applied by itself, but potentiated ACh-elicited current of both α7 and α1β1δε receptors (Figure 5). In terms of peak current amplitude, Hmg 1b-2 application induced a 50% increase of the of α7 nAChR current and a comparatively lower and non-significant enhancement of the α1β1δε current (Figure 5a,b).

### 2.4. Electrophysiological Effects of Hmg 1b-2 Met_ox_ and Hmg 1b-5 on ASIC Channels

**Figure 6:**
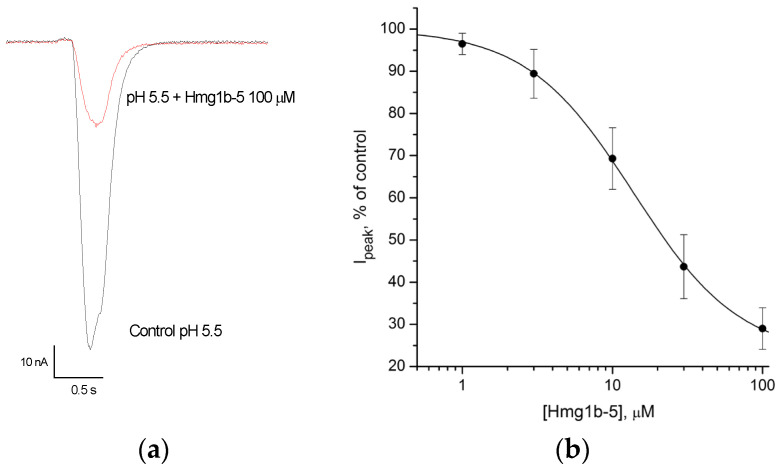
Inhibitory activity of peptide Hmg 1b-5 toward rASIC3 channels. (**a**) Acid-induced currents through rASIC3 expressed in *X. laevis* oocytes were evoked by pH drop from 7.4 to 5.5; the effect of Hmg 1b-5 at a concentration of 100 μM on the transient currents. (**b**) Concentration-response curve for rASIC3 expressed in *X. laevis* oocytes indicating the inhibitory effect of Hmg 1b-5. Each point corresponds to means ± SD (*n* = 5). Data were fitted by a logistic equation. The resulting values of the fitting parameters are: IC_50_ 13.8 ± 0.6 μM, n_H_ of 1.22 ± 0.04, and I_minimal_ 22.4 ± 1.3.

The toxins Hcr 1b-2 (= Hmg 1b-2) and Hcr 1b-4 (= Hmg 1b-4) were previously shown to modulate ASIC1a and ASIC3 channels [16,17]. The ability of new toxins, Hmg 1b-2 Met_ox_ and Hmg 1b-5, to modulate ASIC channels was assessed on homomeric rat (r) ASIC1a and ASIC3 channels expressed in *X. laevis* oocytes. Inward current was induced by a rapid pH drop from 7.4 to 5.5. The investigated peptides were applied 15 s before the acidic pulse. In contrast to Hmg 1b-2 [16,17], the derivative peptide Hmg 1b-2 Met_ox_ demonstrated no effect on rASIC1a or rASIC3 currents. Peptide Hmg 1b-5 inhibited the rASIC3 transient current in a concentration-dependent manner with IC_50_ 13.8 ± 0.6 μM (Figure 6). Inhibition of the acid-induced current by the peptide was not complete and a maximal inhibitory effect at 100 μM Hmg 1b-5 reached about 78%. The activity of Hmg 1b-5 toward rASIC1a was negligible. At maximal applied concentration of 100 μM, it caused 17% inhibition. Thereby only Hmg 1b-5 had inhibitory activity specific to rASIC3.

### 2.5. Sequence Identification and Analysis of H. magnifica APETx-like Toxins Diversity

To gain insight into diversity of APETx-like toxins, the structure and organization of their encoding sequences were characterized using 3′- and 5′-RACE strategy. As a result of Step-Out 3′-RACE with the two forward ASIC_SIGN and ASIC_F primers, seven different cDNAs (~300 bp) containing the stop codon (TAA), polyadenylation site (AATAAA), and poly(A) tract were obtained (Figure S1a). These 3′-RACE sequences were used to design gene-specific primers, ASIC_R1 and ASIC_R2, for Step-Out 5′-RACE. Thirteen different cDNAs (~300 bp) coding a 5′-untranslated region, a signal peptide, a propeptide with a furine proteinase site, and an N-terminal fragment of a mature peptide were obtained (Figure S1a). Then 5′- and 3′-RACE cDNAs were assembled, and full-length cDNAs of ~600 bp were obtained only for Hmg 1b-2 and Hmg 1b-5 (Figure S1b).

**Figure 7:**
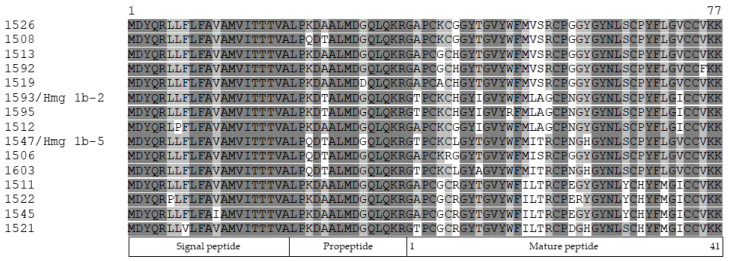
Full-length APETx-like precursors from *H. magnifica.* Identical and conserved amino acid residues are shown on a dark and light gray background, respectively. Vector NTI Advance ™ 11.0 (Invitrogen, Carlsbad CA, USA) [23] was used for multiple sequence alignment.

To determine HmgTx isoforms diversity, two gene-specific primers, APETx_amp_For and APETx_amp_Rev, were designed based on the 5′- and 3′-RACE cDNAs sequences. Cloning and sequencing of ~350 bp PCR products allowed the determination of transcripts encoding 15 different HmgTx precursor proteins. All deduced amino acid sequences consist of a signal peptide (21 aa), a propeptide (13 aa), and a mature peptide (41 aa) (Figure 7). Based on comparative analysis, HmgTx 1593 and 1547 are identical to the natural toxins, Hmg 1b-2 and Hmg 1b-5, respectively.

At the result of comparison of other HmgTx isoforms, amino acid substitutions were observed in the signal peptide region (L/P, F/V, and V/I), propeptide region (K/Q, and A/T), and in the mature peptide chain at positions 2, 5, 7, 10, 14, 16–19, 22–24, 29, 31, 34, 36, 39 (Figure 7). Thus, the HmgTx sequences are characterized by conservative signal peptides, propeptides, and variable mature peptides, which are characteristic features of multigene family representatives [24].

This fact directly indicates the existence of a multigene family of APETx-like peptides (class 1b [25]) in the sea anemone *H. magnifica*. To confirm the presence of several genes, we carried out PCR with *H. magnifica* genomic DNA and the same primers, APETx_amp_For and APETx_amp_Rev, followed by cloning and sequencing of PCR products (~900–1250 bp). At least five different groups of *HmgTx* genes were found which shared the exon–intron–exon structure, in which the intron fell on the propeptide encoding region. The *HmgTx* genes varied in an intron length (from 501 bp up to 853 bp), whereas exons sizes were particularly conserved (unpublished data). The structure of the *Hmg 1b-5* gene was inferred by similarity with HmgTx 1547 cDNA (Figure S2). We were not able to find any other matching gene–cDNA–protein pairs.

The deduced sequences of mature peptides have a high sequences identity (76–100%) with natural APETx-like peptides Hcr 1b-1, 2, 3, and 4 from *H. crispa* [16,22]. To clarify the phylogenetic relationships between *H. magnifica* and *H. crispa*, we sequenced 18S rRNA (18S ribosomal RNA, ~1500 bp), ITS gene region (internal transcribed spacer, ~700 bp), and COI gene (mitochondrial cytochrome c oxidase subunit 1, ~600 bp) of *H. magnifica* 91 and *H. crispa* 116 samples and compared with the same sequences from the GenBank. Based on the phylogenetic analysis, both *H. crispa* 116 and *H. magnifica* 91 belong to the Stichodactylidae family and fall into the *H. magnifica* group (Figure S3). Therefore, the sea anemones previously identified by morphology as *H. crispa* belong to the species *H. magnifica*.

### 2.6. Molecular Modeling of APETx-like Toxins

The HmgTx have sequence identity (41 to 48% on Figure 2) to APETx2, which served as a valid template for 3D structures homology modeling in the case of HcrTxs [17]. The reliable models for each HmgTxs based on the 3D structure of APETx2 (PDB ID: 1WXN [26]) were generated as described in the Materials and Methods section.

The structure–activity relationship indicated that the molecular surface of toxin through which the dipole emerges (basic and hydrophobic patch) is involved in the interaction with ion channels, in particular ASICs [16,27].

**Figure 8:**
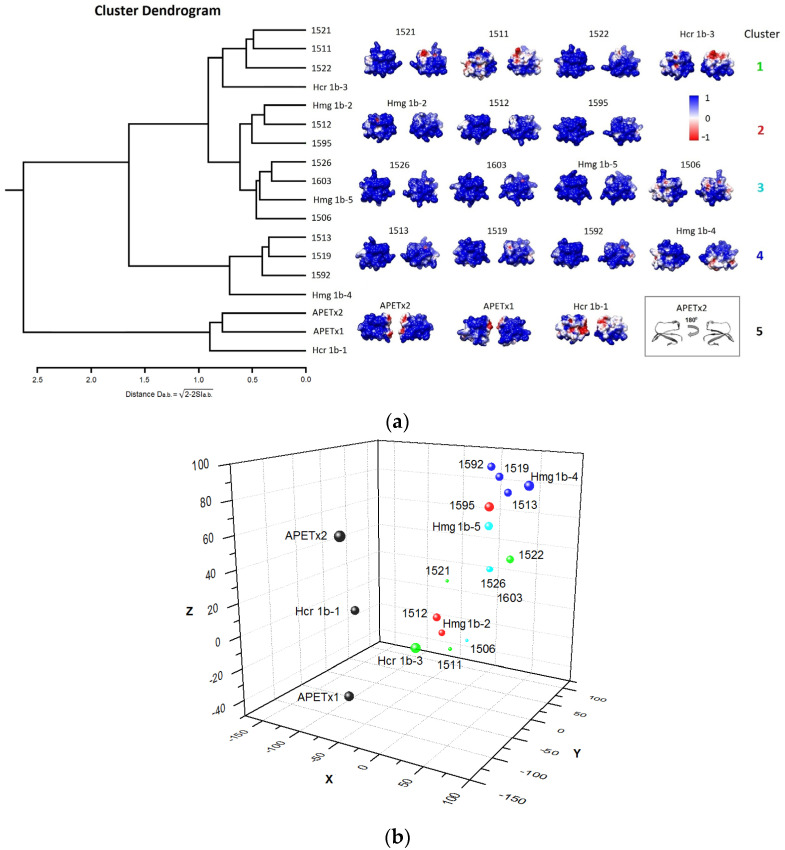
(**a**) PIPSA clustering (**left**) and molecular electrostatic potential surfaces (**right**) of the APETx-like toxins from *H. magnifica* and APETx1 and APETx2 from *A. elegantissima*. The horizontal axis of the dendrogram represents electrostatic similarity distance. Representation of the EP calculated with Delphi web server projected on the molecule solvent accessible surface with Chimera. EP were calculated using ionic strength corresponding to 150 mM salt concentration, colored with blue and red corresponding to color key and presented in two orientations shown in a box as APETx2 molecule ribbon representation. (**b**) Dipole moment direction of APETx-like peptides. 3D scatter plot of the coordinate of superimposed molecules dipole moments. The spheres are colored according to PIPSA clustering and their size corresponds to the dipole moment magnitude from 44 D for HmgTx 1506 up to 148 D for APETx2. Dipole moments were calculated using Discovery studio 4.0 Visualizer (Accelrys Software Inc, San Diego, CA, USA), plot was generated using ORIGIN 7.5 software (OriginLab Corporation, Northampton, MA, USA).

To evaluate how subtle variations in the primary structure influence the electrostatic properties, the models of APETx-like peptides from *H. magnifica* were compared with the functionally characterized homologous APETx1 and APETx2. The electrostatic surfaces of all APETx-like peptides exhibited large strongly negative patches with the few positive moieties (Figure 8a). For the surface electrostatic potential (EP), a similarity quantification dendrogram was generated with the PIPSA web server [28] and the peptides were divided into five clusters. Surprisingly, Hcr 1b-1 grouped together with *A. elegantissima* toxins and demonstrated a maximum of electrostatic similarity distance to homologs from *H. magnifica*. Another intriguing observation was cluster 4, where three toxins grouped with Hmg 1b-4. Whereby these peptides may possess effects on ASICs such as Hmg 1b-4 (i.e., ASIC3 potentiation and ASIC1a inhibition [17]). 

Regarding the dipole moment orientation, the majority of toxins may be conditionally divided into two groups, in reference to dipole moment orientation of 1521, which is located “in the middle” (Figure 8b). The first group consists of Hmg 1b-2, 1506, 1511, 1512, and Hcr 1b-3, and the second includes Hmg 1b-5, 1513, 1519, 1522, 1526, 1592, 1595, and Hmg 1b-4. Three toxins, Hcr 1b-1, APETx1, and APETx2, are distinct from all others as well as from each other and form a separate group. Remarkably, only members of clusters 4 (1513, 1519, 1592, Hmg 1b-4) and 5 (APETx1, APETx2, Hcr 1b-1) are grouped on a plot similar to PIPSA clustering, while toxins of clusters 1–3 with worse electrostatic similarity distance are mixed up.

## 3. Discussion

Both nicotinic receptors and ASIC channels are widely distributed throughout the nervous systems and contribute to synaptic transmission, neuronal excitability, cognitive function, pain signal transduction, etc. They are related to neurodegenerative disorders and other pathological conditions, namely, Alzheimer’s and Parkinson’s diseases, epilepsy, anxiety, depression and addictive behavior. Therefore, nAChRs and ASICs are promising targets for novel neuroprotective and antidepressant drugs [19,20,29,30]. Some clinically used drugs, such as amiloride and tetracaine, have been shown to display cross-reactivity with nAChRs and ASICs and this phenomenon is currently under investigation [31,32].

Here, for the first time we determined that APETx-like toxins of the sea anemone *H. magnifica*, HmgTxs, could compete with the [^125^I]-αBgt for binding to membrane preparations from the electric organ of *T. californica* containing the muscle-type nAChR, and to GH_4_C_1_ cells expressing human α7 nAChR. Among them were two known toxins Hmg 1b-2 and Hmg 1b-4 identical to Hcr 1b-2 and Hcr 1b-4 [16], and two new toxins Hmg 1b-2 Met_ox_ and Hmg 1b-5 which target muscle-type nAChRs. At a concentration of 20 µM HmgTxs showed the following efficiency of competition with [^125^I]-αBgt for binding to muscle-type receptor from *T. californica* membranes: Hmg 1b-2 ≈ Hmg 1b-2 Met_ox_ < Hmg 1b-5 ≈ Hmg 1b-4. The affinities to human α7 nAChR were at least twice as low (40 µM of toxins) with the following order of effectiveness: Hmg 1b-2 Met_ox_ < Hmg 1b-2 ≈ Hmg 1b-4 ≈ Hmg 1b-5. Derivative toxin Hmg 1b-2 Met_ox_ showed the weakest inhibitory activity to human α7 nAChR, but still retains the ability to bind with both receptors. Notably, scorpion toxin HelaTx1 derivative with an oxidized Met was almost as active as the native toxin on *T. californica* nAChR and human α7 nAChR [11].

For the electrophysiological experiments, toxin Hmg 1b-2 (=Hcr 1b-2) was chosen. This toxin was studied by us not only as ASICs’ inhibitor [16], but it also displayed an exceptional lack of selectivity, from the 28 tested voltage gated cation channels, comprising 16 potassium (K_V_), 9 sodium (Na_V_), and 3 calcium (Ca_V_) channels, 26 of them were subject to a certain degree of activation or inhibition by this toxin [33]. Despite such promiscuity to ion channels, this toxin exhibited an antihyperalgesic effect in the model of acid-induced muscle pain [16] and weak anxiolytic activity in the open field and elevated plus maze tests, wherein it did not demonstrate any toxic action or stimulating effects on the central nervous system [34].

Here, we observed that Hmg 1b-2 showed a low affinity (tens of µM) to the muscle-type and non-muscle nAChR subtypes in competition with the [^125^I]-αBgt for binding to orthosteric sites (Figure 2e,f). In *X. laevis* oocytes, Hmg 1b-2 at micromolar concentration proved to be a positive modulator of both human α7 and muscle-type α1β1δε receptors. It potentiated ACh current of α7 nAChR by 50% (Figure 5), while the enhancement of α1β1δε current was not statistically significant. It should be noted that using ACh concentrations above the EC50 value may obscure the true potentiating activity of competitive agonists. Therefore, future experiments using ACh concentrations around the EC50 value (approximately 5 µM Ach [35]) for α1β1δε are needed to investigate more in detail the potentiating activity of Hmg 1b-2 on α1β1δε receptors. It is still unclear if Hmg 1b-2 is an allosteric, orthosteric or combined ligand, but its effects on cholinergic response could be compared with the action of positive allosteric modulators such as α-conotoxin MrIC, and protein lynx-1 [36,37,38]. It should be noted that, based on our previous observation of the potentiating and inhibitory effects of APETx-like peptides on their targets [16,17,22,33], both agonists and antagonists might be found among these toxins.

According to electrophysiological testing, Hmg 1b-2 Met_ox_ had no modulatory effect on ASIC1a and ASIC3 channels, while Hmg 1b-2 inhibited both ASIC1a and ASIC3 channels [16,17]. As far as we know there is no data describing the effect of Met oxidation on the activity of ASICs modulators. According to the molecular modeling of Hcr 1b-2 interaction with ASIC1a, Met16 faces away from channel and does not make intermolecular contact [17]. In the same way as Hmg 1b-2 and Hcr 1b-3 [16,17], toxin Hmg 1b-5 was shown to be an inhibitor of both ASIC1a and ASIC3 channels. However, the activity of Hmg 1b-5 toward ASIC1a was negligible. This distinguishes it from previously characterized HcrTxs which exhibit higher (Hmg 1b-2, Hcr 1b-3) or equal (Hcr 1b-4) activity and efficiency for ASIC1a channels in comparison with ASIC3 [16,17].

The APETx-like peptides of *H. magnifica* share 34–41% of identity with BDS toxins, 41–56% with APETx1–APETx4 and demonstrate substantially higher identity to each other, from 66 to 98%. The remarkable feature of HmgTxs is the dyad of positively charged residues, mostly Lys40-Lys41, at the C-termini of peptide that was not observed for BDS or APETx1–APETx4. In addition, positively charged residues are commonly localized close to N-termini (positions 5 or 7) and at position 19. Moreover, most HmgTxs do not contain negatively charged residues. Accordingly, HmgTxs are very basic peptides (pI from 8.65 to 9.78). Moreover, molecules Hmg 1b-5, 1603, 1595, 1526, 1506, 1592, 1519, and 1522, have an extended positive potential which is way beyond the molecular surface of the peptide along one side of the molecule. Given that some HmgTxs target ASICs, this is an important characteristic since the molecular surface of ASICs channels is negatively charged, especially the acidic pocket, the putative binding site of APETx-like peptides [17]. Based on the electrostatic properties of the APETx-like peptides, we assumed that it is unlikely that any of novel *Heteractis* peptides share the orientation of APETx2 molecule in complex with ASIC channel. Moreover, if our previous hypothesis considering various hot-spot interactions of Hcr 1b-2 and Hcr 1b-4 with ASIC1a is correct, it is reasonable to assume that there are some other possible architectures of complexes between ASIC channels and homologous peptides with unique electrostatic characteristics. This might explain the reduced efficiency of ASIC1a inhibition (in comparison with ASIC3) observed for Hmg 1b-5, a novel peptide with extended positive potential. In contrast, previously investigated peptides Hcr 1b-2–Hcr 1b-4 effectively inhibit ASIC1a.

Proteinaceous toxins and neuropeptides affecting ASICs were isolated from spiders, snakes, sea anemones, wasps, and cone snails [39,40]. There is a single report describing conorfamides from the Mexican cone snail *Conus austini* that inhibit non-neuronal α7 and muscle-type nAChRs at nanomolar concentrations as well as modulate homomeric ASIC1a and ASIC3 at micromolar concentrations, altering channel desensitization. Interestingly, conorfamides are linear peptides unlike disulfide-bonded α-conotoxins which are well known as nAChRs ligands [41]. According to the data obtained in this study, APETx-like peptides could be added to this list. It was not surprising as they are a clear illustration of the sea anemone toxins characterized by functional promiscuity [42,43]. The first identified APETx-like peptides, BDS toxins and APETx1, were initially reported to target voltage-gated potassium channels, K_V_3.4 and hERG (human ether-a-go-go related gene), respectively [44,45]. Further electrophysiological investigation, however, showed the opposite effects of BDS-I on tetrodotoxin-sensitive (TTX-sensitive) and TTX-resistant sodium currents as well as inhibition of mammalian voltage-gated sodium channels (Na_V_) by APETx1 [46,47]. APETx3 differs from APETx1 only by Thr3Pro substitution, and only inhibited the inactivation of arthropods and TTX-sensitive mammalian Na_V_ channels [46]. The first toxin from sea anemones, which is an inhibitor of ASIC3, is the peptide APETx2 [48], which also inhibited hERG and Na_V_ channels [46,49]. Finally, peptide APETx4 was an inhibitor of several Na_V_ and K_V_ channels including the subtype hEag1 [50]. The APETx-like peptides Hcr 1b-1–Hcr 1b-4 turned out to be the modulators of ASIC1a and ASIC3 channels [16,17], while Hcr 1b-2 inhibited in sum 23 subtypes of Na_V_, K_V_, and Ca_V_ channels as well as potentiate K_V_1.1, K_V_1.2, and Shaker channels [33], as noted above.

The peptides from venomous animals, including sea anemones, have been known to form combinatorial libraries encoded by multigene families. We have reported Kunitz-type peptide libraries [21,24,51], as well as an actinoporins library of *H. crispa* [52,53] and now we have data that prove the existence of a combinatorial library of APETx-like peptides of *H. magnifica*. Remarkably, the proteome and/or transcriptome studies resulted in finding APETx-like peptides in sea anemones belonging to the family Actiniidae, such as *A. viridis* [54], *Anemonia sulcata* [55], *Cnidopus japonicus* [56], *A. elegantissima* [57], and *Bunodosoma granulifera* [58], but not in sea anemones from Stichodactylidae family, such as *Stichodactyla haddoni* [59], *Stichodactyla duerdeni* [60], and *Stichodactyla helianthus* [58]. Here, we pointed out the existence of an APETx-like peptide combinatorial library in *H. magnifica* encoded by the *HmgTxs* multigene family. Considering that we did not find all matching gene–cDNA–protein pairs, more experiments are needed to fully explore the structural diversity of peptide isoforms in the sea anemone *H. magnifica*.

## 4. Conclusions

We have determined that APETx-like toxins from the sea anemone *H. magnifica* are new ligands to muscle-type and α7 nAChR subtypes. The promiscuous toxin Hmg 1b-2, acting on ASICs and voltage-gated cation channels, also selectively potentiates the ACh-elicited current of the α7 but not muscle-type nAChRs. Oxidation of Met16 in Hmg 1b-2 was indicated to abolish its inhibitory effect on ASICs channels and radioligand displacement in case of the α7 nAChR but not muscle-type nAChRs. A novel peptide Hmg 1b-5, unlike its homologues, inhibits ASIC3 and has negligible effect on ASIC1a channels. We showed that HmgTxs form an APETx-like peptide combinatorial library encoded by the *HmgTxs* multigene family that makes this sea anemone a promising source of novel multifunctional toxins. These toxins could be seen as tools for the investigation of functional activity of different ion channels as well as prototypes for the molecular design of pharmacologically active agents. Additionally, we demonstrated the importance of a combination of morphological and phylogenetic determination of biological organisms for scientific research; since specimens, previously morphologically described as different species, are characterized as *H. magnifica* in this study.

## 5. Materials and Methods

The specimens of *H. magnifica* were collected from the South China Sea near Tho Chu islands, Vietnam (09°19.3 N; 103°29.6 E), during a marine expedition aboard the research vessel Academic Oparin in 2010. Dr. E.E. Kostina (A.V. Zhirmunsky Institute of Marine Biology, National Scientific Center of Marine Biology Far Eastern Branch, Russian Academy of Sciences, Vladivostok, Russia) confirmed the identity of the species.

nAChR-enriched membranes from the electric organs of *Torpedo californica* ray were kindly provided by Prof. F. Hucho (Free University of Berlin, Berlin, Germany). GH4C_1_ cells transfected with human α7 nAChR were a gift from Eli-Lilly. Radiolabeled 125I-α-buhgarotoxin with specific radioactivity of 500 Ci/mmol was prepared as in [61]. A-Cobratoxin was purified from *Naja kaouthia* venom as described in [62].

The study was conducted according to the guidelines of the Convention on Biological Diversity, and approved by the Ethics Committee of the G.B. Elyakov Pacific Institute of Bioorganic Chemistry (Vladivostok, Russia, Protocol No. 0037. 12 March 2021).

### 5.1. Extraction and Chromatographic Procedure

Sea anemones were put into the aquarium with sea water and not fed for a week, then they were used once for mucus extraction with milking technique [63]. For this, they were placed in a plastic bag where their tentacles were massaged to obtain mucus.

Peptide fraction was then separated by hydrophobic chromatography on polychrome-1 (powdered Teflon, Biolar, Olaine, Latvia) column (4.5 cm × 14 cm). Hydrophobic peptides eluted with 40% aqueous ethanol were separated by size exclusion chromatography in an automatic FPLC system (ÄKTApurifier^®^, GE Healthcare, Uppsala, Sweden) using a Superdex Peptide 10/30 column. The peptides were eluted with 10% acetonitrile in 0.1% trifluoroacetic acid (TFA) at a flow rate of 0.1 mL/min. The protein concentration was determined by the Lowry method [64], bovine serum albumin was used as a standard, and using the absorbance at 280 nm. After the size exclusion chromatography active fraction was separated by HPLC on a reversed-phase Luna C18 column (10 mm × 250 mm) equilibrated with 10% acetonitrile solution in 0.1% TFA on an Agilent 1100 chromatograph (Agilent Technologies, Santa Clara, CA, USA). Peptides elution was carried out using a gradient of acetonitrile concentration (with 0.1% TFA and at a flow rate of 1 mL/min): 10% for 10 min, then 10–70% for 60 min. The further separation of the active peptide fractions and final purification of major peptides were made on the same column in two gradients of acetonitrile concentration (with 0.1% TFA and at a flow rate of 1 mL/min): 10% for 5 min, 10–40% for 30 min, then 40% and 10% for 5 min, 10–40% for 20 min, then 40%. Vacuum concentrator 5301 (Eppendorf Inc., Hamburg, Germany) was used for acetonitrile evaporation.

### 5.2. Reduction and Alkylation of Disulfide Bridges

Peptides were reduced and alkylated with 4-vinylpyridine as described in [65]. Separation of the reaction mixture was made on a reversed-phase Nucleosil C18 column (4.6 mm × 250 mm) equilibrated with 10% acetonitrile in 0.1% TFA. The elution was carried out using gradient of acetonitrile concentration (with 0.1% TFA and at a flow rate of 0.5 mL/min), 10% of acetonitrile for 30 min, 10–70% for 60 min.

### 5.3. Cyanogen Bromide Cleavage of Alkylated Peptides

The reaction was carried out in 70% TFA at room temperature for 4 h in the dark. The molar ratio of cyanogen bromide:peptide was 100:1. Separation of the reaction mixture was made on reversed-phase Nucleosil C18 column (4.6 mm × 250 mm) equilibrated with 10% acetonitrile in 0.1% TFA. The elution was carried out using a combined gradient of acetonitrile concentration at a flow rate of 0.5 mL/min, 10% of acetonitrile for 30 min, 10–70% for 60 min.

### 5.4. Mass Spectrometric Analysis

A mass spectrometric analysis was carried out using an Ultraflex TOF/TOF mass spectrometer (Bruker Daltonik, Karlsruhe, Germany). The samples were solved in acetonitrile/water solution (1:1, *v*/*v*) containing 0.1% TFA and mixed with 10 mg/mL sinapinic acid as a matrix. Protein molecular masses (1000–20,000 Da) were obtained in linear mode with external calibration.

### 5.5. Tandem Mass Spectrometry (MS/MS), Sequence Determination and Analysis

The amino acid sequences were identified from the collision-induced dissociation (CID) tandem mass spectra of peptide fragments obtained by the cyanogen bromide cleavage of the peptide molecule previously treated with 4-vinylpyridine. The CID MS/MS experiments were performed on an ultra-high resolution quadrupole time-of-flight mass spectrometer MaXis impact (Bruker Daltonik, Karlsruhe, Germany) equipped with an ESI ionization source. A survey mass spectrum and tandem mass spectrum were recorded for each sample. During MS/MS, the fragment ions were generated from the isolated multiple charged precursor ion of peptide fragments. The precursor ions were fragmented by low-energy CID with collision energy from 30 eV to 85 eV.

The sequences identity was analyzed using amino acid sequence databases and the BLAST algorithm (http://www.ncbi.nlm.nih.gov/BLAST (accessed on 5 September 2022)) [66]. Multiple alignment of amino acid sequences was made using Vector NTI Advance ™ 11.0 (Invitrogen, Carlsbad, CA, USA) (https://www.thermofisher.com/ru/ru/home/life-science/cloning/vector-nti-software.html (accessed on 15 December 2008)) [23]. The theoretical calculation of isoelectric point was performed using the software GPMAW-Lite (https://www.alphalyse.com/customer-support/gpmaw-lite-bioinformatics-tool (accessed on 5 September 2022)). The protein sequence data of Hmg 1b-5 reported in this paper will appear in the UniProt Knowledgebase under the accession number C0HLS4.

### 5.6. cDNA and Gene Sequences Determination, Phylogenetic Analysis

Genomic DNA was extracted from sea anemone tissues using the MagJET Plant Genomic DNA Kit (Thermo Fisher Scientific, Waltham, MA, USA). Total RNA was isolated from intact RNA fixed-tentacle samples using ExtractRNA solution (Evrogen, Moscow, Russia). Full-length-enriched cDNA libraries were prepared from the total RNA using Mint cDNA synthesis Kit (Evrogen, Moscow, Russia). The rapid amplification of cDNA 3′-ends (3′-RACE) was carried out with the RACE primer set (Evrogen, Moscow, Russia) and the forward primers ASIC_sign (5′-TCTTTGATTGCAGCTTC-3’) on Step 1, and ASIC_F (5´-AAGCGTGGAACASMTTG- 3´) on Step 2. The primers were designed based on the known signal sequences of APETx-like peptides from the sea anemones of the genus *Actiniidae* (UniProtKB/Swiss-Prot G0W2H8.1, G0W2H9.1, G0W2I0.1) [58], as well the N-terminal amino acid sequences of native peptides modulating ASIC channels of sea anemone *H. crispa* [16,22]. 5′-RACE was performed with the RACE primer set (Evrogen, Moscow, Russia) and the reverse primers ASIC_R1 (5´-AAGTAGGGGCAAGAGAGA-3´) and ASIC_R2 (5´-CATRARCCAGTAGACACC-3´), designed based on obtained 3`-RACE sequences. The cDNA and gene sequences were amplified using PCRs with APETx_amp_For (5`-AATCCAATCCAAACACGGCCAT-3`) and APETx_amp_Rev (5`-AGTTGTTTGG GTCAGATTCTTGTCA-3`) created based on obtained 3′ and 5′-RACE sequences. All PCRs were conducted with Encyclo^®^ DNA Polymerase (Evrogen, Moscow, Russia). All primers were synthesized by Evrogen (Moscow, Russia).

PCR-fragments were analyzed by gel electrophoresis, purified, cloned into pTZ57R/T using T/A cloning system (Thermo Fisher Scientific, Waltham, MA, USA), and transformed into DH5α *E. coli* cells (Thermo Fisher Scientific, Waltham, MA, USA) according to standard protocols. PCR products from positive colonies were sequenced with M13 universal primers using the ABI 3130xl Genetic Analyzer (Applied Biosystems, Foster City, CA, USA).

The 18S rRNA and COI genes, and ITS gene region were amplified and sequenced as described in [67]. Gene sequences were deposited in GenBank under accession numbers ON797294 (for *H. magnifica* 116) and ON926908 (for *H. magnifica* 91) for the 18S rRNA genes; ON797309 (for *H. magnifica* 116) and OP107886 (for *H. magnifica* 91) for the COI gene; ON831386-ON831387 (for *H. magnifica* 116) and ON936908-ON936909 (for *H. magnifica* 91) for the ITS genes region.

The 18S rRNA, COI, and ITS sequences were aligned by MEGA X software version 11.0.9 [68] using Clustal W algorithm with sea anemone sequence homologs searched in the GeneBank database (http://www.ncbi.nlm.nih.gov/BLAST, accessed on 24 June 2022). Phylogenetic analysis was conducted using MEGA X 10.2 software [68]. Phylogenetic trees were constructed on model-tested alignments according to the maximum likelihood algorithm. The topologies of the trees were evaluated by 1000 bootstrap replicates.

### 5.7. Radioligand Competition Assay

In the competition experiments with [^125^I]-αBgt, investigated fractions and purified peptides (at chosen concentration) were pre-incubated for 3 h at room temperature with *T. californica* electric organ membranes (final concentration 1.25 nM of toxin-binding sites) or with the GH_4_C_1_ cells (6.5 μg of total protein with final concentration of 0.4 nM of toxin-binding sites) in 50 μL of binding buffer (20 mM Tris-HCl buffer, 1 mg/mL of bovine serum albumin, pH 8.0). After that, [^125^I]-αBgt was added to the membranes or GH_4_C_1_ cells to a final concentration of 0.4 nM and the mixtures were additionally incubated for 5 min. The binding was stopped by rapid filtration on GF/C filters (Whatman, Clifton, N.J., USA) pre-soaked in 0.25% polyethylenimine, the unbound radioactivity having been removed from the filters by washout (3 × 3 mL) with a binding buffer. Non-specific binding was determined in all cases using 3 h pre-incubation with 30 μM α-cobratoxin.

### 5.8. Expression of nAChRs in Xenopus laevis Oocytes and Electrophysiological Recordings

For the expression of nAChRs (adult rat muscle-type α1β1δε and human α7 nAChRs) in *Xenopus* oocytes, the linearized plasmids were transcribed to RNA using the T7 or SP6 mMESSAGEmMACHINE transcription kit (Ambion, Austin, TX, USA). Mature female animals were purchased from Nasco (Fort Atkinson, WI, USA) and housed at KU Leuven Aquatic Facility in compliance with the regulations of the European Union (EU) concerning the welfare of laboratory animals, as declared in Directive 2010/63/EU. The use of *X. laevis* oocytes was approved by the Animal Ethics Committee of the KU Leuven with the license number P186/2019. Stage V–VI oocytes were collected from female *X. laevis* frog as previously described [69], with the frogs anesthetized by placement in 0.1% tricaine solution (amino benzoic acid ethyl ester; Merck, Kenilworth, NJ, USA). Oocyte microinjection was performed using a microinjector (Drummond Scientific^®^, Broomall, PA, USA), with a programmed cRNA injection volume of 50 nL. The concentration of the injected RNAs ranged from 700–1500 ng/µL. The oocytes were incubated at 16 °C in ND96 solution (96 mM NaCl; 2 mM KCl; 1.8 mM CaCl_2_; 2 mM MgCl_2_ and 5 mM HEPES, pH 7.4), supplemented with 50 mg/L gentamicin sulfate (Panpharma GmbH, Hameln, Germany) and 180 mg/L theophylline (Sigma-Aldrich, St Louis, MO, USA).

Electrophysiological measurements were performed at room temperature (18–22 °C) using the two-electrode voltage-clamp (TEVC) technique, 2–5 days after injection. Data were sampled at a frequency of 100 Hz and low-pass filtered at 20 Hz by a four-pole Bessel filter, using a GeneClamp 500 amplifier (Axon Instruments^®^, Burlingame, CA, USA), and Clampex9 software (Axon Instruments^®^, Burlingame, CA, USA). Glass micropipettes were produced using glass capillaries (borosilicate WPI 1B120-6) and drawn in a WPI (World Precision Instruments^®^, Sarasota, FL, USA) manual stretcher. The bath and perfusion solution was the previously described ND96.

Cells were clamped at a holding potential of −70 mV and continuously perfused with ND96 buffer. Current responses were evoked by applying 100 µM (α7) or 500 µM (muscle-type α1β1δε) ACh (Sigma-Aldrich, St Louis, MO, USA) solubilized in ND96 buffer, under gravitational flow, until peak current amplitude was observed. A control of 3 pulses of ACh was carried out, with 30 s of interval between each pulse. Hmg 1b-2 (1 µM) was applied directly in the perfusion chamber, without gravitational flow, and incubated for 60 s. Afterwards, a new ACh pulse was applied. Peak current amplitudes was measured prior to and following the incubation of the peptide.

The differences in channel activity between the control and toxin conditions were compared by a one-way ANOVA followed by Tukey’s multiple comparisons test, using the software GraphPad Prism 8.0.2. Differences were considered statistically significant when *p* < 0.1.

### 5.9. Expression of ASIC Channels in Xenopus laevis Oocytes and Electrophysiological Recordings

Rat ASIC1a and ASIC3 channels were expressed in *X. laevis* oocytes after injection of 2.5–10 ng of cRNA, as previously described [70]. After injection oocytes were kept for 2–3 days at 19 °C and then up to 5 days at the temperature of 15–16 °C in sterile ND96 medium (96 mM NaCl, 2 mM KCl, 1.8 mM CaCl_2_, 1 mM MgCl_2_, 5 mM HEPES titrated to pH 7.4 with NaOH supplemented with 50 μg/mL of gentamycin). Two-electrode voltage clamp recordings were performed using GeneClamp 500 amplifier (Axon Instruments, Burlingame, CA, USA). The data were filtered at 50 Hz and digitized at 1000 Hz by an AD converter L780 (LCard, Moscow, Russia) using in-house software (Moscow). The solutions were applied to a cell chamber (volume 50 μL). The laminar flow of an external solution of ND96 (pH 7.4) was used at a rate of 1 mL/min. ASIC1a and ASIC3 were activated by a short (1 s) application of a solution with a pH 5.5 (10 mM MES) using a fast application system. Peptides were applied 15 s before the activation pulse in a solution containing 0.1% BSA. A value of currents inhibition was calculated as the ratio of the peak current amplitude, when the peptide was applied to the average amplitude of the control peak currents before and after the peptide application and expressed as a percentage. To construct the concentration-response curves, a logistic equation was the following: y = ((1 − A)/(1 + ([C]/IC_50_)^nH^)) + A; where y is the relative value of current inhibition; C is the peptide concentration; IC_50_ is the half maximal inhibitory concentration; n_H_ is the Hill coefficient; A is an amplitude of maximum inhibition (% of control).

### 5.10. Homology Modeling

Homology models of the 3D structure of APETx-like peptides from *H. magnifica* were constructed using Chimera 1.11.2rc software [71] with Modeller 9.19 plug-in [72] based on spatial structures of APETx2 (PDB ID 1WXN and 2MUB) [26,49]. Five models of each HmgTxs were generated and those based on 1WXN 3D structure were chosen for feather analysis as the most reliable because of minimal root mean square deviation (RMSD) values and backbone conformations occupying “allowed” regions of the Ramachandran plot. EP calculations were made with the Delphi web server [73], and results were visualized in the Chimera interface. Web PIPSA software [28] was used for clustering analysis.

## Data Availability

Not applicable.

## Supplementary Materials

The following supporting information can be downloaded at: https://www.mdpi.com/article/10.3390/toxins14100697/s1, Figure S1: (a) 3`- and 5` RACE translated sequences. (b) Full-length cDNAs of Hmg 1b-2 and Hmg 1b-5. Amino acid substitutions are shown by green (Hmg 1b-2) and yellow (Hmg 1b-5) colors; Figure S2: Structure of *HmgTx 1b-5* gene inferred by similarity of 42.27 gene sequence with HmgTx 1547 cDNA. The signal pep-tide region is highlighted by orange, the propeptide region is highlighted by green, and the mature chain is high-lighted by blue. The furine proteinase site is colored by red. The mature peptide is highlighted by blue; Figure S3: ML trees showing phylogenetic relationships of the sea anemone *H. magnifica* 91 (present study) and *H. magnifica* 116 (as *H. crispa* 116 in previous study [16]) in the Stichodactylidae family based on sea anemone COI gene sequences (a), 18S gene sequences (b), and ITS gene sequences (c). Sea anemones *Anthopleura elegantissima*, *Actinia equina*, *Anthopleura artemisia*, *Actinia bermudensis*, *Bunodosoma caissarum*, and *Anthopleura atodai* belong to the Actiniidae family. Bootstrap values (%) of 1000 replications. Nodes with confidence values greater than 50% are indicated.
