# Supplementary material for: Nicotinic Acetylcholine Receptors Are Novel Targets of APETx-like Toxins from the Sea Anemone Heteractis magnifica
Source: Toxins (Basel). 2022 Oct 11;14(10):697. doi: 10.3390/toxins14100697 (PMC9611977; doi:10.3390/toxins14100697)
Supplement: Supplementary file 1 [file toxins-14-00697-s001.zip › toxins-1927212-supplementary.pdf]

toxins

MDPI

Article

Nicotinic Acetylcholine Receptors Are Novel Targets of APETx-like Toxins from the Sea Anemone *Heteractis magnifica*

Rimma S. Kalina, Igor E. Kasheverov, Sergey G. Koshelev, Oksana V. Sintsova, Steve Peigneur, Ernesto Lopes Pinheiro-Junior, Roman S. Popov, Victoria E. Chausova, Margarita M. Monastyrnaya, Pavel S. Dmitrenok, Marina P. Isaeva, Jan Tytgat, Sergey A. Kozlov, Emma P. Kozlovskaya, Elena V. Leychenko and Irina N. Gladkikh

```
108          MDYQRLLFLFAVAMVITTTVALPQDTALMDGQLQKRGAPCKCGGYTGVYWLM
110          MDYQRLLFLFAVAMVITTTVALPQDTALMDGQLQKRGAPCKCGGYTGVYWFM
106          MDYQRLLFLFAVAMVITTTVALPKDAALMDGQLQKRGAPCKCGGYTGVYWFM
109          MDYQRLLFLFAVAMVITTTVALPKDAALMDGQLQKRGAPYKCGGYTGVYWLM
1078         MDYQRLLFLFAVAMVITTTVALPKDAALMDGQLQKRGAPCKCGGYTGVYWLM
111          MDYQRLLFLFAVAMVITTTVALPKDAALMDGQLQKRGAPCGCHGYTGVYWLM
107          MDYQRLLFLFAVAMVITTTVALPKDAALMDGQLQKRGAPCGCHGYTGVYWFM     5` RACE
1081         MDYQRLLFLFAVAMVITTTVALPKDAALMDGQLQKRGAPCGCRGYTGVYWLM
1097         MDYQRLLFLFAVAMVITTTVALPQDTALMDGQLQKRGTPCKCLGYTGVYWFM
102          MDYQRLLFLFAVAMVITTTVALSKDTALMDGQLQKRGTPCKCHGYIGVYWFM
104          MDYQRLLFLFAVAMVITTTVALPKDTALMDGQLQKRGTPCKCHGYIGVYWFM
1082         MDYQRLPFLFAVAMVITTTVALPKDTALMDGQLQKRGTPCKCHGYIGVYWFM
1077         MDYQRLLFLFAVAMVITTTVALPKDTALMDGQLQKRGTPCKCHGYIGVYWLM
996          ----------------------------------------------GVYWFMLAGCPNGYGYNLSCPYFLGICCVKK
993/Hmg 1b-4          -------------------------------------GVYWFMLSRCPSGYGYNLSCHYFMGICCVKR
1203         ----------------------------------------------GVYWFMLSRCPNGHGYNLSCPYFLGVCC---
992          ----------------------------------------------GVYWFMITRCPNGHGYNLSCPYFLGVCCVKK     3` RACE
994          ----------------------------------------------GVYWFMVSRCPGGYGYNLSCPYFLGVCCVKK
1217         ----------------------------------------------GVYWFMVSRCPGGYGHNLSCPYFLGVCCVKK
916          ----------------------------------------------GVYWLMVSRCPGGYGYNLSCPYFLGVCCVKK
```

(a)

```
Hmg 1b-2                                                 GTPCKCHGYIGVYWFMLAGCPNGYGYNLSCPYFLGICCVKK
104 5`RACE       MDYQRLLFLFAVAMVITTTVALPKDTALMDGQLQKRGTPCKCHGYIGVYWFM
996 3`RACE                                                         GVYWFMLAGCPNGYGYNLSCPYFLGICCVKK
1593             MDYQRLLFLFAVAMVITTTVALPKDTALMDGQLQKRGTPCKCHGYIGVYWFMLAGCPNGYGYNLSCPYFLGICCVKK

Hmg 1b-5                                                 GTPCKCLGYTGVYWFMITRCPNGHGYNLSCPYFLGVCCVKK
1097 5`RACE      MDYQRLLFLFAVAMVITTTVALPQDTALMDGQLQKRGTPCKCLGYTGVYWFM
992 3`RACE                                                         GVYWFMITRCPNGHGYNLSCPYFLGVCCVKK
1547             MDYQRLLFLFAVAMVITTTVALPQDTALMDGQLQKRGTPCKCLGYTGVYWFMITRCPNGHGYNLSCPYFLGVCCVKK
```

(b)

Figure S1. (**a**) 3`- and 5` RACE translated sequences. (**b**) Full-length cDNAs of Hmg 1b-2 and Hmg 1b-5. Amino acid substitutions are shown by green (Hmg 1b-2) and yellow (Hmg 1b-5) colors.

```
1547                                       M  D  Y  Q  R  L  L  F  L  F  A  V  A  M  V  I  T  T  T  V  A  L  P  Q  D  T  26
42.27 gene     TAATCCAATC CAAACACGGC CATGGATTAT CAAGACTCCT CTTCCTGTTT GCTGTTGCCA TGGTGATTAC AACAACTGTA GCACTTCCTC AGGATACTGC 100
1547 cDNA      TAATCCAATC CAAACACGGC CATGGATTAT CAAGACTCCT CTTCCTGTTT GCTGTTGCCA TGGTGATTAC AACAACTGTA GCACTTCCTC AGGATACTGC 100
               ---------------------→
1547           A  L  M  D  G                                                                                            31
42.27 gene     CTTGATGGAT GGTAATGGAA CTTTACTCGA CATGTTATGC GCTTGGTTAT GCCGTGATTC CTTCAGGTCC CTCTCCCCCC TTCCCCCCCA CCTTACACAC 200
1547 cDNA      CTTGATGGAT GGT------- ---------- ---------- ---------- ---------- ---------- ---------- ---------- ---------- 113
42.27 gene     ACACACACAC ACAATACGTA CCTCTGTTTC TTCCGCCTTA CAATAAAATG ACTTCATGTG AGTCATTTGC GCTCCGCTTA GCGCGTGTTT ACAAGAGGGC 300
1547 cDNA      ---------- ---------- ---------- ---------- ---------- ---------- ---------- ---------- ---------- ---------- 113
42.27 gene     AGTTTTCTGC TTGGCCACGT ATATGTAATA ATTTAAGGTA ATGGTAAAGA TCGATTTGGC ACTGGAAAAT AAAATCAAAC ATATGAAGAA TCTCTATTCC 400
1547 cDNA      ---------- ---------- ---------- ---------- ---------- ---------- ---------- ---------- ---------- ---------- 113
42.27 gene     TCCCTCCTCT CCCTTCCAAC AATCTAAACG CTTTACTCGA CTTATATTCA TTTTTAAAAT TAGTAAAAGG ACATTTTATT TATGATGTTT GGACGTGTTT 500
1547 cDNA      ---------- ---------- ---------- ---------- ---------- ---------- ---------- ---------- ---------- ----------
42.27 gene     GTAGGTAATT ATAATTCGTT GTCTTGTTAC TGTACTGTTG TCAAGTACTC TTTTCTTGCA TTTTGAGCGT TCAGTGTTTT GTAGTGTCTG ACTGACATAG 600
1547 cDNA      ---------- ---------- ---------- ---------- ---------- ---------- ---------- ---------- ---------- ----------
42.27 gene     CACACTTGAT AACCTTGATA TCATTCTACA CACTTATCCA TCACTCAGTC ATGGACTAGA AATAATGGAA AGTTATAGTG CTTAGAAAGG AGGTTATTCC 700
1547 cDNA      ---------- ---------- ---------- ---------- ---------- ---------- ---------- ---------- ---------- ----------
42.27 gene     GGGCGAAAGA CCCTTGGAAA TAAATTCGGA AAAATTAATT TCCGACCTCG CTGGGATTCG AACCCACGAC CTTCGGATTT GTAGTACCGA CGCTCTACCA 800
1547 cDNA      ---------- ---------- ---------- ---------- ---------- ---------- ---------- ---------- ---------- ----------
42.27 gene     GCTGAGCTAC AGGTCAGACT GGGAGCAGGC CGGGGTGAAA TAGATGTTAC GTCGCGGTAA TTTTATGTGC TACATATTCA TATTCATATT CACATCACTC 900
1547 cDNA      ---------- ---------- ---------- ---------- ---------- ---------- ---------- ---------- ---------- ----------
1547                                                                                        Q  L  Q  K  R  G  T  P  C  K  C  L  45 (7)
42.27 gene     AGTCATTTAT GCGTATGATT AGTTCTAGTT ATTAAGGCTT TACTTACTTT TTTTTCCCTT CAGGTCAACT GCAAAAACGC GGAACGCCTT GTAAGTGCCT 1000
1547 cDNA      ---------- ---------- ---------- ---------- ---------- ---------- -----CAACT GCAAAAACGC GGAACGCCTT GTAAGTGCCT 148
1547             G  Y  T  G  V  Y  W  F  M  I  T  R  C  P  N  G  H  G  Y  N  L  S  C  P  Y  F  L  G  V  C  C  V  K  88 (40)
42.27 gene     TGGTTATACT GGTGTTTACT GGTTTATGAT CACACGTTGT CCAAATGGAC ACGGATACAA TCTCTCTTGC CCCTACTTTT TGGGGGTTTG CTGTGTTAAA 1100
1547 cDNA      TGGTTATACT GGTGTTTACT GGTTTATGAT CACACGTTGT CCAAATGGAC ACGGATACAA TCTCTCTTGC CCCTACTTTT TGGGGGTTTG CTGTGTTAAA 248
1547           K Stop                                          89 (41)
42.27 gene     AAATAAATCG GATTGACGAT GTGACAAGAA TCTGACCCAA ACAACTA  1247
1547 cDNA      AAATAAATCG GATTGACGAT GTGACAAGAA TCTGACCCAA ACAACTA   295
                                    ←------------------------
```

Figure S2. Structure of *HmgTx 1b-5* gene inferred by similarity of 42.27 gene sequence with HmgTx 1547 cDNA. The signal peptide region is highlighted by orange, the propeptide region is highlighted by green, and the mature chain is highlighted by blue. The furine proteinase site is colored by red. The mature peptide is highlighted by blue.

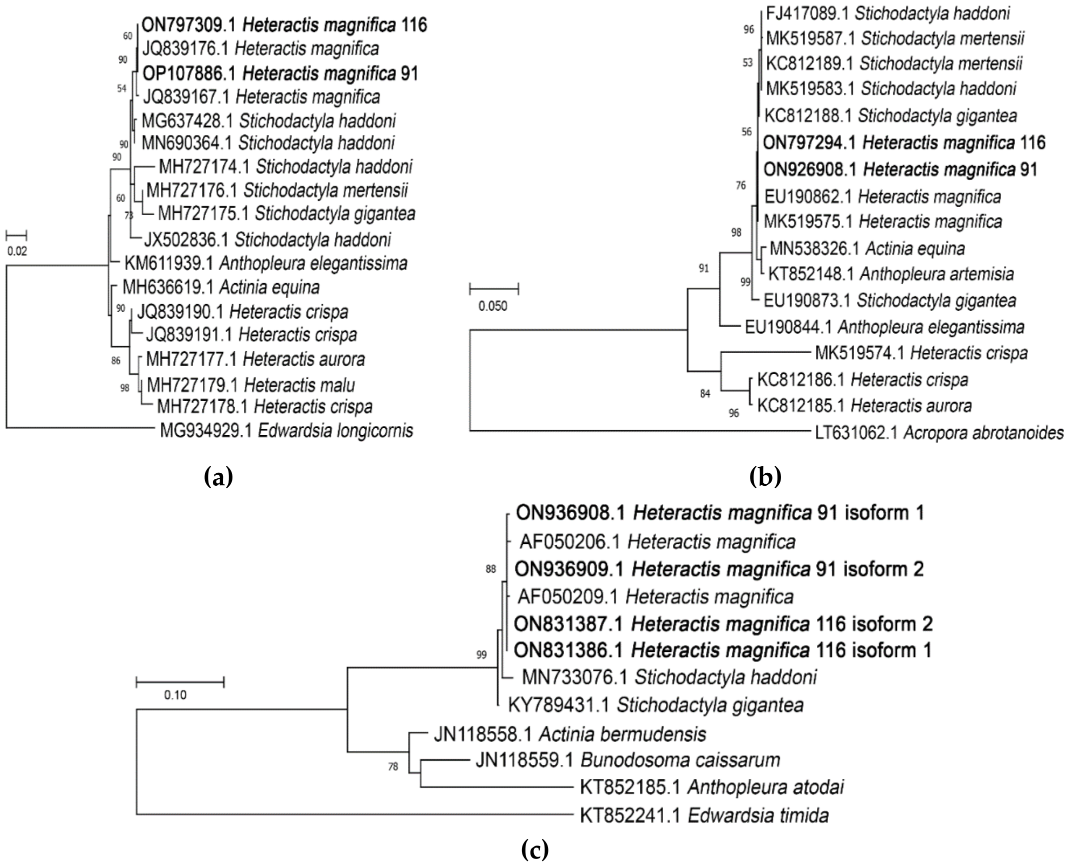

Figure S3. ML trees showing phylogenetic relationships of the sea anemone *H. magnifica* 91 (present study) and *H. magnifica* 116 (as *H. crispa* 116 in previous study [16]) in the Stichodactylidae family based on sea anemone COI gene sequences (**a**), 18S gene sequences (**b**), and ITS gene sequences (**c**). Sea anemones *Anthopleura elegantissima*, *Actinia equina*, *Anthopleura artemisia*, *Actinia bermudensis*, *Bunodosoma caissarum*, and *Anthopleura atodai* belong to the Actiniidae family. Bootstrap values (%) of 1000 replications. Nodes with confidence values greater than 50% are indicated.
